# Advances in Human Mitochondria-Based Therapies

**DOI:** 10.3390/ijms24010608

**Published:** 2022-12-29

**Authors:** Gang Zhong, Jagadeesh K. Venkatesan, Henning Madry, Magali Cucchiarini

**Affiliations:** Center of Experimental Orthopaedics, Saarland University Medical Center, Saarland University, Kirrbergerstr. Bldg 37, 66421 Homburg, Germany

**Keywords:** mitochondria, aging, oxidative disorders, inflammatory diseases, mitochondrial diseases, cancer, regenerative medicine

## Abstract

Mitochondria are the key biological generators of eukaryotic cells, controlling the energy supply while providing many important biosynthetic intermediates. Mitochondria act as a dynamic, functionally and structurally interconnected network hub closely integrated with other cellular compartments via biomembrane systems, transmitting biological information by shuttling between cells and tissues. Defects and dysregulation of mitochondrial functions are critically involved in pathological mechanisms contributing to aging, cancer, inflammation, neurodegenerative diseases, and other severe human diseases. Mediating and rejuvenating the mitochondria may therefore be of significant benefit to prevent, reverse, and even treat such pathological conditions in patients. The goal of this review is to present the most advanced strategies using mitochondria to manage such disorders and to further explore innovative approaches in the field of human mitochondria-based therapies.

## 1. Introduction

The symbiotic state of mitochondria and eukaryotic cells traces back to a ‘‘survival of the fittest’’ event that occurred about 1.5 billion years ago, when a prokaryotic cell engulfed an alpha-probacteria and retained it as a functional component, namely an organelle [1]. Mitochondria inherit the structural and genetic basis of their bacterial ancestors, consisting of two separate, functionally distinct outer mitochondrial membranes (OMMs) and inner mitochondrial membranes (IMMs) that enclose intermembranous spaces (IMs) and a stromal compartment possessing a partially independent circular genome—the mitochondrial DNA (mtDNA)—reduced during evolution by gene transfer to the nucleus [2,3,4]. Over a long period of natural adaptation and evolution, newly acquired intracellular symbionts produced new specialized aerobic respiration that efficiently extracts energy from glucose through the electron transport chain (ETC) and oxidative phosphorylation (OXPHOS) to produce adenosine triphosphate (ATP), the main ‘‘currency’’ that provides energy metabolism for living organisms [5]. This overwhelms the original energy operating rules of eukaryotic cells, becoming the main energy pathway, and is supported by the bacteriophage-related mitochondrial maintenance systems, supported by the observation that the closest relatives of many mtDNA modifying enzymes (such as mtDNA polymerases) are bacteriophage proteins [4].

The long-term intracellular symbiont state facilitates in-depth communication and compromise between mitochondria and eukaryotic cells that allows the intracellular grinding into a dynamic, interconnected network tightly bound to other cellular compartments. Mitochondria, as the central node in the operation of this system, are not only powerpacks of the cells but also perform a smooth shuttle with the nucleus, making the mitochondria–nucleus information delivery crucial for the maintenance of the intracellular metabolism in physiological conditions that may be affected during pathological situations including aging, oxidation, inflammation, immunological diseases, metabolic syndromes, obesity, cancer, and degenerative disorders. The mitochondrial retrograde signaling is an important way to affect the decision-making of the nucleus accomplished by retrograde signal-mediated protein activation of nuclear gene expression or by altering its epigenetics through DNA methylation and post-translational modification of histones [6,7]. The nucleus responds to signals from the mitochondria to assist cellular metabolic crises under the various pathological conditions listed above. In addition to having a strong network with the nucleus, mitochondria are equally tightly connected with other organelles and the membrane contact sites between them are critical for lipid and ion exchange, membrane dynamics, and signal transduction [8]. For example, mitochondria and lysosomes cooperate with each other to complete autophagy [9]. Mitochondrial binding to the endoplasmic reticulum (ER) via mitochondria-associated ER membranes (MAMs) [10] controls the transport of calcium, a regulator of the overall mitochondrial membrane potential (∆Ψm) and an important second messenger that transmits external or cytoplasmic signals to the mitochondria, and is affected in neurodegenerative diseases and during aging [11,12].

**Figure 1:**
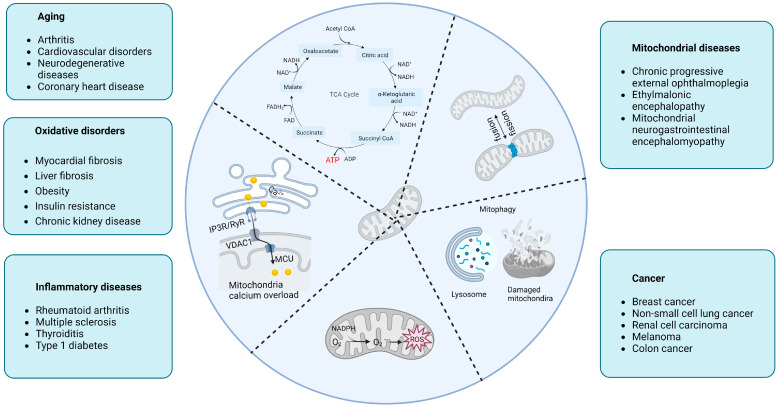
Central roles of mitochondria in human diseases. Abbreviations: CoA, coenzyme A; NADH, nicotinamide adenine dinucleotide; ATP, adenosine triphosphate; ADP, adenosine diphosphate; FADH2, flavine adenine dinucleotide; TCA, tricarboxylic acid; MCU, mitochondrial calcium uniporter; VDAC1, voltage-dependent anion channel 1; IP3R, inositol 1,4,5-trisphosphate receptor; RyR, ryanodine receptor.

Mitochondria play an important role in the normal functioning of cells, either as an energy supply station or as a metabolic information hub. Mitochondrial dysfunction emerged to be implicated in a growing number of major diseases common in humans, such as aging, oxidative disorders, inflammatory diseases, mitochondrial diseases, and cancer (Figure 1).

To address this fundamental problem, a number of therapeutic strategies have been developed to target the mitochondria in these human pathologies and also in the field of regenerative medicine as presented thereafter.

## 2. Strategies Targeting Mitochondria in Aging

Aging is by far the greatest risk factor for a variety of common human diseases including cardiovascular disorders, arthritis, coronary heart disease, and neurodegenerative diseases. Mitochondria have long been implicated in aging, with a time-dependent accumulation of mtDNA variants and an imbalance of reactive oxygen species (ROS) [13]. Most of these aging-related disorders are first associated with shattered energy-demanding organs such as the myocardium, brain, and muscle, where energy is supplied by mitochondria performing the OXPHOS pathway to conduct electrons in the ETC for a conversion of electrical potential energy into bioenergy that supports cellular activities [14]. In senescent cells, electron transport in the ETC is blocked and leaks into the mitochondrial matrix [15]. This undesired electron robs the mitochondrial IM of protons, depleting the ΔΨm, which is extremely important for electron transport in the ETC and reacts with oxygen to generate ROS [16] causing irreversible damage to DNA (mtDNA and nuclear DNA), proteins, and biological membranes [17].

**Figure 2:**
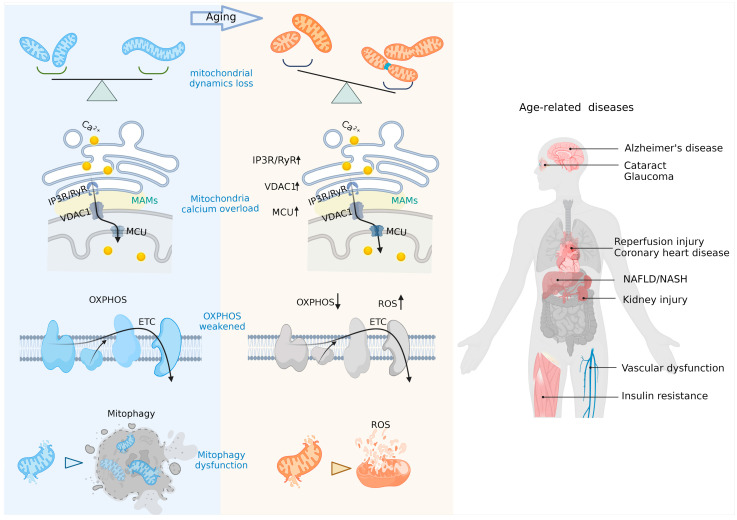
Mitochondrial biology in age-related disorders. Abbreviations: MAMs, mitochondria-associated endoplasmic reticulum membranes; MCU, mitochondrial calcium uniporter; VDAC1, voltage-dependent anion channel 1; IP3R, inositol 1,4,5-trisphosphate receptor; RyR, ryanodine receptor; OXPHOS, oxidative phosphorylation; ETC, electron transport chain; NAFLD, non-alcoholic fatty liver disease; NASH, non-alcoholic steatohepatitis.

Due to their role as multifaceted regulators of aging and cellular senescence (Figure 2), mitochondria have therefore been targeted to generate anti-aging treatments by balancing mitochondrial metabolism and mitophagy (the “quality control” mechanism of mitochondria breaking down damaged mitochondria and removing dysfunctional and undesirable mitochondrial components and by-products), via maintaining mitochondrial calcium (mitoCa^2+^) homeostasis, and modulating mitochondrial dynamics [18,19,20,21,22,23,24,25,26,27,28,29,30,31,32,33,34,35,36,37,38,39] (Table 1).

Mitochondrial metabolic alterations from OXPHOS to glycolysis are frequently observed in senescent cells [40,41], whereas defending the efficiency of OXPHOS from being impeded was shown to be beneficial for the lifespan of living organisms [42], suggesting that active repair and reversal of mitochondrial metabolism may be valuable to tackle aging. Specifically, Din et al. [18] demonstrated that overexpressing regulators of mitochondrial biogenesis (cellular Myelocytomatosis—c-myc, peroxisome proliferator activated receptor gamma coactivator 1 alpha—PGC-1α, proviral insertion in murine 1—Pim-1) in a mouse model of cardiomyocyte aging was capable of reversing cardiac aging by maintaining mitochondrial function. Other gene regulation targets may include pumilio2 (PUM2), 6-phosphofructo-2-kinase/fructose-2,6-bisphosphatase 3 (PFKFB3), activating transcription factor 3 (ATF3), sirtuin 3 (SIRT3), sirtuin 4 (SIRT4), and nuclear enriched abundant transcript 1 (NEAT1) [22,23,24,25,26,27,28]. In addition, administering biomodulators may provide effective, alternative therapeutic approaches capable of modulating mitochondrial functions in response to aging such as coenzyme Q (CoQ), an important component in ETC receiving electrons from complex I and complex III [43]. CoQ-deficient ETCs frequently appear in senescent cells and CoQ supplementation may optimize mitochondrial functions by normalizing ∆Ψm and enhancing ATP synthesis in senescent cells [44]. Mitoquinone (MitoQ) or O-(3-piperidino-2-hydroxy-1-propyl)nicotinic amidoxime (BGP-15) have been proven to modulate the ∆Ψm and ROS contents to reverse aging-associated meiotic spindle defects in mice and humans [45]. Weimer et al. [32] also reported that D-glucosamine (GlcN) is able to increase mitochondrial respiration by promoting the dependence of energy metabolism on OXPHOS while impairing glycolysis to contribute to an extension of the lifespan in many biological species (nematodes, aged mice).

Calcium overload in mitochondria is an important sign of aging [46]. The ER is an important source of mitoCa^2+^ through MAMs which achieve spatial and functional coupling of mitochondria with the ER via the inositol 1,4,5-trisphosphate receptor (IP3R)-glucose-regulated protein 75 (Grp75)-voltage-dependent anion channel (VDAC) [47]. In senescent cells, IP3R and VDAC are overactivated, resulting in unconstrained calcium flux into mitochondria from the ER [48,49] where calcium concentrations are 5- to 10-fold higher than in the mitochondria [50]. The resulting calcium overload further triggers abnormal ∆Ψm and elevated ROS [51], thus accelerating the aging process in a “vicious circle”. Limiting calcium overload may therefore be another important approach to suppress aging. For instance, Wiel et al. [52] showed that inhibiting the IM mitoCa^2+^ uniporter holocomplex (MCU) membrane protein (the direct channel of calcium in the mitochondria) prevented a persistent accumulation of mitoCa^2+^ and that knocking down IP3R2 (the inositol 1,4,5-trisphosphate receptor type 2—ITPR2—and main channel of calcium exchange on MAMs) can reduce ROS levels and inhibit senescence. Other significant factors to counterbalance aging by inhibiting mitoCa^2+^ include the tumor suppressor candidate 2 (Tusc2/Fus1), ATF3, CDGSH iron sulfur domain 2 (Cisd2), isradipine, verapamil, and α-Klotho [24,53,54,55,56,57].

As dynamic cell organelles, the mitochondria perform a continuous cycle of fission and fusion referred to as mitochondrial dynamics. This process ensures proper mitochondrial functions in response to nutrient demands, signal transduction, and external stress [58]. Abnormal mitochondrial dynamics is compromised in aging and related disorders, as evidenced by the occurrence of an aberrant (giant) mitochondrial morphology [59] and maintaining or restoring the normal mitochondrial dynamics may allow researchers to tackle the aging process in affected cells. For instance, up-regulating the cytosolic dynamin-related protein 1 (DRP1—a central protein that coordinates mitochondrial fission by mediating membrane contraction and fission) [60] can prolong the lifespan of Drosophila by facilitating mitophagy and improving mitochondrial respiratory functions [38,61]. Other proteins that may be targeted include mitochondrial fission factor (MFF), fission protein-1 (FIS1), mitochondrial Rho 1 (MIRO1), mitochondrial dynamics protein-49 (MiD49), and mitochondrial dynamics protein-51 (MiD51), all involved in the mitochondrial dynamic networks and deviate from physiological expression during aging, by normalizing them in cells in order to restore natural mitochondrial functions, morphology, and lifespan in living organisms [62].

## 3. Strategies Targeting Mitochondria in Oxidative Disorders

Oxidative stress recapitulates a state of unbalanced antioxidant effects due to excessive ROS levels leading to oxidative damage in the body that affects various intracellular biomolecules such as DNA, proteins, and lipids in the course of aging and other human disorders [63]. As a major ROS producer, the mitochondria that play crucial roles in the cellular emergency responses (oxidative stress, physical stimulation, calcium overload) are the first systems attacked [64], resulting in loss of membrane elasticity, disruption of ∆Ψm, in mtDNA mutations, and reduced ATP synthesis efficiency leading to mitochondrial dysfunction [65]. Mitochondria-targeted antioxidant therapy to reduce excessive ROS levels may therefore be an effective means to diminish or even prevent oxidative damage in affected cells and organs. Such a strategy by ROS scavenging and by the pharmacological manipulation of mitochondrial biogenesis is based on the use of antioxidant moieties, of gene therapy, and of traditional Chinese medicine (TCM) monomers with antioxidant potency.

Since mitochondrial oxidative damage involves lipid peroxidation and as the binding of alkyl polypropylenes to the IMMs strongly exacerbates its damage [66], initial research first focused on the use of antioxidants that are effective against lipid peroxidation. Ubiquinone [67], tocopherol [68], lipoic acid [69], the peroxidase mimetic ebselen (MitoPrx) [70], and their derivatives have been extensively reported for their effectiveness against mitochondrial oxidative damage. MitoQ that is reduced by the complex II to an active ubiquinone antioxidant in the respiratory chain has been also particularly validated by driving the ∆Ψm and lipid peroxidation in numerous clinical trials [71]. In addition, the superoxide dismutase mimetic M40403 (MitoSOD) (resist O_2_^•−^), MitoPrx (resist H_2_O_2_), TEMPOL (MitoTEMPOL) (resist OH•), vitamin E (MitoE) (resist OH•), and lipoic acid (MitoLip) (resist O_2_^•−^, H_2_O_2_ and OH•) are additional antioxidants recognized for their ROS scavenging effects [72]. To increase mitochondrial targeting, lipophilic cations such as triphenylphosphonium (TPP) that can bind to antioxidants and allow their passage through the mitochondrial membrane [73,74,75], were introduced in a follow-up attempt, greatly (100-fold) enhancing mitochondrial antioxidant uptake [76].

Regarding potential gene therapy interventions, several antioxidant genes were described to regulate the mitochondrial metabolism and to block oxidative stress by regulating OXPHOS metabolites. Among them, the mitochondrial signal transducer and activator of transcription 3 (STAT3) that manipulates the complex Ⅰdehydrogenase activity through a retrograde nicotinamide adenine dinucleotide (NAD^+^) signal may improve cellular antioxidant activities [77]. In addition to targeting OXPHOS metabolites, other gene regulation strategies may further suppress oxidative stress. For instance, the use of the hypoxia-inducible factor alpha (HIF-1α), a master regulator of tissue responses to oxidative pathological stimuli, may modulate damaging ROS levels that impair cardiac function in myocardial fibrosis after myocardial infarction [78]. Suppressive effects of HIF-α on mitochondrial oxidative stress were also documented for the treatment of liver fibrosis [79], obesity [80,81], and insulin resistance [82]. In addition, overexpression of the neurofibromatosis-1 (NF1) was shown to advantageously increase resistance to oxidative and mitochondrial respiration while reducing the levels of ROS production by 60% in Drosophila melanogaster through adenylyl cyclase (AC)/cyclic adenosine monophosphate (cAMP)/protein kinase A (PKA) signaling [83].

The application of TCM has been performed for more than 2000 years and in recent years, various TCM monomers have been reported in pharmacological research as effective antioxidants to treat a number of human diseases. The herbal salvia miltiorrhiza has been widely used for cardiovascular diseases as it can balance the production of ROS in cardiomyocytes [84,85] and reduce the oxidative damage of cardiac ischemia-reperfusion [86,87]. The anti-inflammatory and anti-tumoral Atractylodes lactone extracted from the rhizome of Atractylodes macrocephala Koidz can counteract the oxidative stress associated with chronic kidney disease, reducing muscle wasting via inhibition of the phosphoinositide 3-kinase (PI3K)/protein kinase B (PKB or AKT)/mammalian target of rapamycin (mTOR) pathway [88]. Other beneficial TCM compounds may include curcumin, an inhibitor of oxidative stress and mitochondrial dysfunction in astrocytes [89], cuscuta pedicellata extract that improves oxidative stress caused by high-quality diet through gene protection [90], and artemisinin protecting glutamate-mediated neuronal oxidative apoptosis by manipulating the ∆Ψm and reducing ROS levels [91].

## 4. Strategies Targeting Mitochondria in Inflammatory Diseases

Growing evidence supports the contribution of mitochondrial dysregulation to an inflammatory phenotype in numerous diseases such as rheumatoid arthritis [92], multiple sclerosis [93], thyroiditis [94], and type 1 diabetes [95]. With aging or upon tissue damage, the mitochondria release undesired tricarboxylic acid metabolites and damaged mitochondrial components (mtDNA, cardiolipin, N-formyl peptides) recognized as damage-associated molecular patterns (DAMPs) that act as danger signals to trigger the immune system via pattern recognition receptors (PRRs) [96]. For instance, circulating mtDNA gradually increases after 50 years of age [97] correlating with an enhanced production of pro-inflammatory cytokines such as in cultured monocytes [97] and in elderly individuals [97]. Succinate, a tricarboxylic acid metabolite, induces HIF-1-mediated interleukin 1 beta (IL-1β) production [98] and its accumulation promotes reverse electron transport from complex Ⅱ to complex Ⅰ, a pattern resulting in dramatic elevation of ROS levels [99]. Other mitochondrial metabolites with immunostimulatory effects include citrate, acetate, acetyl-CoA, and itaconate contributing to inflammation and immune responses [100]. When stimulated by pathogen-associated molecular patterns (PAMPs) and DAMPs, MAMs are also involved in sensitive molecular signals by providing sites for the activation of the inflammasome as large protein complexes controlling the activation of the proteolytic caspase 1 enzyme. In turn, this regulates the maturation of IL-1β and IL-18 and cell death (pyroptosis via the formation of gasdermin D-mediated lytic pores in the plasma membrane), for instance the nucleotide oligomerization domain (NOD)-like receptor protein 3 (NLRP3) inflammasome [101]. Owing to the importance of mitochondria in the development of inflammation, several mitochondria-targeted strategies have been explored as anti-inflammatory therapeutic options focusing on mitophagy, ROS control, and on repressing the inflammasome.

The removal of dysfunctional and undesirable mitochondrial components such as mtDNA and ROS by mitophagy contributes to inflammation as DAMPs that are sensed by inflammatory signals [96]. Interestingly, there is evidence that mitophagy combined with the mitochondrial unfolded protein response (UPRmt) can significantly mitigate LPS-mediated inflammatory myocardial injury [102] and that the nuclear factor kappa B (NF-κB) and downstream p62 that recognize damaged mitochondria may activate mitophagy, contributing to homeostasis and tissue repair [103]. The protection of mitophagy against chronic inflammation has also been demonstrated in other disorders including colitis [104], diabetes [105], and Alzheimer’s disease [106].

Another promising therapeutic strategy for inflammation is to control the production of ROS, such as using the various approaches and compounds cited (MitoQ, BGP-15, MitoSOD, MitoPrx, MitoTEMPOL, MitoE, MitoLip, HIF-1α, NF1, TCM, etc.) [45,52,72,78,83,84,85,86,87,91,107].

Restricting the inflammasome via mitochondrial regulation may further be beneficial to control inflammatory pathological phenotypes [108] such as during pathogen infection (human immunodeficiency virus—HIV) [109], sepsis [110], colitis [111], particulate matter (PM) 2.5-mediated pulmonary pyroptosis [112], and more recently during the COVID-19 pandemic with its associated severe acute respiratory syndrome (SARS) due to the SARS coronavirus (SARS-CoV) caused by an excessive inflammatory storm led by NLRP3 [113] such as using the MitoTEMPOL antioxidant that can regulate formation of the NLRP3 inflammasome and reduce SARS-CoV viroporin 3a protein-induced IL-1β secretion for instance [114].

## 5. Strategies Targeting Mitochondria in Mitochondrial Diseases

Mitochondrial diseases represent a group of maternally inherited metabolic disorders caused by mutations in mtDNA that lead to mitochondrial dysfunction, mostly affecting OXPHOS and the levels of ATP synthesis [115] together with a loss of enzymatic intermediates, the accumulation of toxic metabolites, and the disruption of the Krebs and folate cycles [116]. Current therapeutic strategies for mitochondrial diseases are divided into small molecule therapy and mtDNA gene editing.

Supplementation with small molecules is currently the most commonly employed treatment modality for mitochondrial diseases as a means to remove toxic compounds, replenish defective enzymes or their analogs, or balance cofactors since toxic metabolites often trigger the clinical phenotype of mitochondrial diseases. For instance, accumulation of hydrogen sulfide in patients with ethylmalonic encephalopathy (a disease caused by mutations in the ethylmalonic encephalopathy protein 1 (ETHE1) gene coding for a sulfur dioxygenase) may be reduced by the combined application of N-acetylcysteine and metronidazole [117,118,119]. In addition, erythrocyte encapsulation of thymidine kinase phosphorylase was reported to improve mitochondrial neurogastrointestinal encephalomyopathy (MNGIE) syndrome in patients, a mitochondrial disease caused by thymidine phosphorylasethymine phosphorylase (TYMP) mutations leading to thymidine phosphorylase deficiency [120,121,122]. A similar approach was applied to primary CoQ deficiency disorders such as encephalonephropathies with nephrotic syndrome, childhood-onset mitochondrial diseases, and isolated cerebellar ataxia using CoQ supplementation [123,124]. Complementary therapy of enzyme cofactors (non-protein small molecules, metal hydrate ions) has also been successfully used such as by supplementation of vitamin B12, a cofactor for flavin mononucleotide (FMN) and flavin adenine dinucleotide (FAD), to treat mitochondrial diseases associated with FMN and FAD deficiencies (complex Ⅰ deficiency due to acyl-CoA dehydrogenase protein 9—ACAD9—mutations, severe X-linked mitochondrial encephalomyopathy due to mutations in the apoptosis-inducing factor mitochondrion-associated 1—AIFM1—precursor with NAD-dependent NADH oxidase activity) [125,126,127,128].

The mtDNA gene editing technology may also offer powerful options to treat mitochondrial diseases [129] based on antigenomic mtDNA therapy or on the use of restriction endonucleases [130,131,132], zinc-finger nucleases (ZFNs) [133,134], transcription activator-like effectors nucleases (TALENs) [135,136,137], and the clustered regularly interspaced short palindromic repeats/CRISPR-associated 9 (CRISPR/Cas9) [138,139,140] to correct mtDNA variants [141,142,143,144,145], especially with the emergence of double-stranded DNA deaminase (DddA)-derived cytosine base editors (DdCBEs) that can support the editing of C•G to T•A in mutated mtDNA sequences [142,143,144,145]. Still, single base editing is challenging due to genome complexity and probably insufficient to fully reverse a diseased phenotype while it may lead to off-target modifications [146], all impacting the clinical translation potential of mtDNA gene editing.

## 6. Strategies Targeting Mitochondria in Cancer

**Figure 3:**
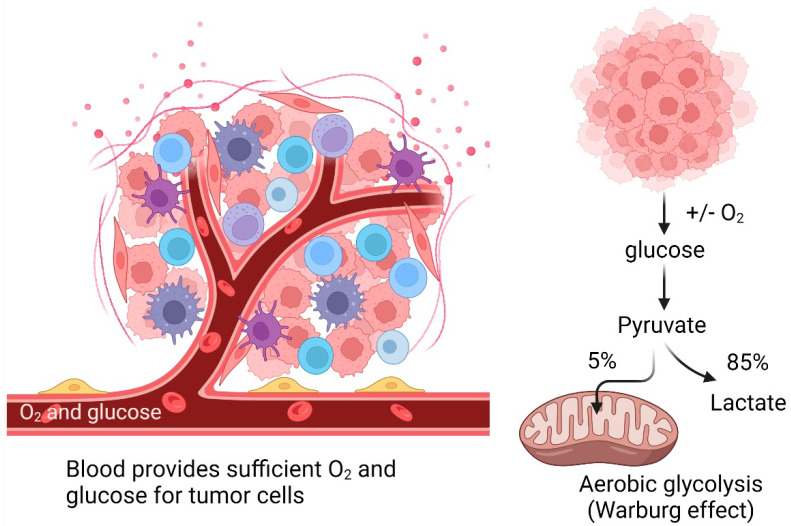
The Warburg effect in cancer.

Mitochondrial damage in tumor cells is associated with an abnormally weakened OXPHOS, supported by the observation that these cells perform the glycolytic pathway to catabolize glucose in lactate to generate ATP even in the presence of sufficient oxygen (Warburg effect) (Figure 3) [147,148] while maintaining high ROS levels [149] that activate molecular signaling pathways promoting tumor cell proliferation [63].

Regulating mitochondrial energy metabolism may therefore be a powerful strategy for tumor therapy and several approaches have been attempted based on the use of specific pathway inhibitors or on the targeting of mitochondrial material metabolism and of mitophagy, including in conjunction with nanodelivery technologies to improve efficacy and reduce adverse reactions [150].

Different inhibitors of critical mitochondrial energy metabolic processes have been applied such as KUNB31 and geldanamycin, two inhibitors of the heat shock protein 90 (HSP90) that block ATP binding and hydrolysis in non-small cell lung cancer, multiple myeloma, ovarian cancer, melanoma, and renal cell carcinoma [151,152] or metformin, an ETC blocker of the complex I that can reduce the production of ATP in breast cancer, non-small cell lung cancer, renal cell carcinoma, melanoma, and colon cancer [153,154].

In addition, as tumor cells need a sufficient material on a regular basis to maintain their rapid proliferation and metabolism, targeting mitochondrial material metabolism presents another attractive treatment modality against cancer. Tricarboxylic acid (TCA) cycle intermediates provide raw materials for the biosynthesis of macromolecules [155] and glutamine is the major carbon source that replenishes TCA cycle intermediates while maintaining their use in biosynthesis in many tumor cells. Glutamine is converted to glutamate by the glutaminase (GLS) for further catabolism to produce beneficial α-ketoglutarate for the cellular material cycle [156]. Blocking this metabolic pathway is therefore an attractive approach in cancer therapy and indeed, multiple specific GLS inhibitors have been developed including the compound 968 and bis-2-(5-phenylacetamido-1,2,4-thiadiazol-2-yl)ethyl sulfide (BPTES), reducing glutamine catabolism and modulating the growth of glutamine-dependent tumors [157,158].

Mitophagy is also involved in the rapid metabolism of tumor cells as another part of the mitochondrial “quality control” process by generating TCA cycle intermediates for tumor cell metabolism [159]. Interestingly, knocking-out the essential autophagy related 7 (ATG7) gene for mitophagy allows increased tolerance to starvation and prolonged lifespan of mice with lung cancer [160]. Chloroquine, another well-known inhibitor of autophagy, and its derivative hydroxychloroquine have also been reported for their excellent tumor suppressive effects [161,162,163]. In addition, given that tumor cells are in a delicate oxidative balance adapted to different ROS levels than normal cells, disrupting this balance with high selectivity has been shown as a potential therapeutic approach for targeting tumors [74,164,165].

## 7. Strategies Targeting Mitochondria for Regenerative Medicine

The repair of damaged tissues is generally supported by the differentiation of progenitor (stem) cells, a critical process associated with pivotal biological activities (protein synthesis, genome replication, carbohydrate preparation) that necessitate large amounts of energy from the mitochondria. However, the contribution of mitochondria to stem cell differentiation is not limited to providing energy but also probably associated with mitochondrial biogenesis and dynamics [166,167]. This is in light of evidence showing that mitochondria are present at higher mass in differentiating cells, with a more elongated morphology, larger surface area, and lower membrane potential compared with their situation in pre-differentiated cells [168,169,170]. Regulating mitochondrial biological behavior may therefore be of strong value to promote stem cell differentiation for tissue repair such as by regulating mitochondrial dynamics (fission, fusion), mitochondrial respiration, and mitoCa^2+^ uptake.

Changes in mitochondrial morphology controlled by mitochondrial dynamics are closely related to the modulation of mitochondrial functions. Owing to the central role of the mitochondria in cellular processes, mitochondrial dynamics is extremely important in the cell cycle at the level of cell proliferation, differentiation, and aging and regulating mitochondrial fission. Fusion is thus an important way to influence the fate of stem cells [171]. Several studies, for instance, reported the significant impact of the dynamin-related protein 1 (Drp1), a molecule directing mitochondrial fission, on the differentiation of eukaryotic cells both in vitro and in vivo. Specifically, ablation of Drp1 in the mouse brain was shown to cause cerebellar hypoplasia in association with the presence of few giant mitochondria in Purkinje cells (instead of normally many short tubular mitochondria) [171] while neural cell-specific Drp1(−/−) mice were reported to develop brain hypoplasia with reduced neurite numbers and abnormal synapse formation, dying shortly after birth [172]. Accordingly, treatment with Drp1 inhibitors such as the mitochondrial division inhibitor 1 (mdivi-1, an inhibitor of the guanosine triphosphate hydrolase (GTPase) activity of Drp1) that prevents myotube formation and impairs the myogenic differentiation of myoblasts [173] or with the mitochondrial fission 1 protein (FIS1) involved in myeloid differentiation [174] may provide strong tools to address these critical problems. On the other side, there is evidence that mitofusin 2 (Mfn2), a receptor located in OMMs and contributing to mitochondrial fusion, plays a key role in mammalian stem cell differentiation and that overexpression of Mfn2 in human-induced pluripotent stem cells (hIPSCs) promotes their differentiation and maturation in neurons [175].

Mitochondrial respiration, in particular the intermediate ROS product, is also important for stem cell differentiation. Interestingly, while ROS is considered detrimental at unrestricted levels, closely regulated ROS levels may have a beneficial impact on cellular functions such as cell proliferation and differentiation [176]. For instance, appropriate ROS levels are capable of enhancing the adipogenic differentiation of mesenchymal stem cells (MSCs) by controlling specific signaling cascades (c-jun NH2-terminal kinase—JNK, p38 mitogen-activated protein kinase—p38 MAPK, extracellular regulated kinase—ERK, phosphatidylinositol 3-kinase/protein kinase B—PI3K/Akt—pathways) in these cells [176]. In contrast, inhibition of mitochondrial respiration via hypoxia and mitochondrial ETC inhibitors was shown to significantly suppress the adipogenic differentiation of MSCs [177]. Other studies demonstrated that the overexpression of superoxide dismutase 2 (SOD2), a mitochondrial antioxidant metalloenzyme, can significantly improve bone differentiation and bone formation in mice with osteogenic differentiation defects by assisting SIRT3 and regulating the mitochondrial oxidative transition and respiratory activities [178]. Finally, as an important intermediate of OXPHOS, providing the main carbon source for macromolecular synthesis and displaying antioxidant properties, glutathione was reported to increase the differentiation capacity of MSCs for bone regeneration [179,180].

Apart from its effects on mitochondrial metabolism, an imbalance in mitoCa^2+^ may also affect the regenerative capacity of cells and tissues [49,181,182]. This concept is supported by the observation that increasing mitoCa^2+^ via inhibition of the mitochondrial calcium uptake 1 (MICU1, the “gatekeeper” of mitoCa^2+^ uptake in the MCU) attenuates the regenerative capacity of liver cells [183]. In contrast, overexpression of MICU1 in a Streptococcus pneumoniae-mediated lung injury model can significantly reduce mitoCa^2+^ uptake and induce AT2 cells to alveolar cell differentiation as a beneficial process to promote alveolar repair [184], while MICU1 can increase α-ketoglutarate by reducing mitoCa^2+^ uptake and influence myofibroblast differentiation [185].

## 8. Conclusions and Perspectives

Mitochondria are complex organelles that control multiple molecular signals and cellular activities via the formation of interactive networks with other organelles and the nucleus to coordinate cellular behavior and to defend cells against external stress factors. Mitochondria play a central role in this signaling network that can receive instructions from the nucleus to focus on cellular tasks such as ATP synthesis. In addition, they can provide feedback information to the nucleus through retrograde signals to initiate a balance mechanism. Mitochondria also cooperate with the ER and lysosomes to complete various cellular functions such as calcium ion conduction, biomembrane flow, and autophagy.

However, when this balance is altered, the functions of the mitochondria become compromised such as during human aging, oxidative disorders, inflammatory and mitochondrial diseases, cancer, and degenerative pathologies as reviewed herein, and the resulting mitochondrial dysfunction will turn this fine signaling network into a vicious circle. Therefore, targeting the mitochondria as candidates for therapy may be a potent strategy to control and manage such human disorders.

As further reported herein, the feasibility of such a therapeutic paradigm has been explored in human medicine and due to an increased knowledge of mitochondria, a number of mitochondria-based therapeutic approaches have been developed using drug therapy, gene regulation, gene therapy, and genetic engineering procedures to tackle mitochondrial dysfunction and manage human mitochondria-related diseases. However, a number of challenges and issues still remain to be critically addressed prior to clinical translation. While the use of cost-effective drugs and compounds may be readily applicable in a clinical situation considering the high numbers of patients affected by these various pathologies, the effects may not be sufficiently robust for an effective, long-lasting treatment without the manifestation of undesirable adverse effects in generally irreversible human pathologies [186,187,188]. On the other hand, strategies that aim at durably or even permanently modulating genetic phenotypes involved in mitochondrial dysfunction are more challenging and invasive than the use of recombinant agents since they are based on the arduous production of complex, mitochondria-specific gene transfer vectors in a consistent, scalable, and globally affordable manner, requiring considerable amounts of research (academic and industrial) funding and being constrained by regulatory agencies [189,190,191,192,193] especially when involving genome editing procedures [129,193]. Alternatively, based on the observation that mitochondrial transfer naturally occurs between cells through tunneling nanotubes (TNTs) and extracellular vesicles (EVs) [194,195,196,197], somatic mitochondrial transfer, and to a broader extent mitochondrial transplantation, emerged as possible exogenous replacement therapies to compensate for the functional deficit of damaged mitochondria in somatic human adult cells such as in disorders of the brain (Alzheimer’s and Parkinson’s diseases, cerebral ischemia), heart (myocardial infarction, cardiomyopathies), lungs (acute distress syndrome, acute lung injury), liver, kidneys (diabetic nephropathy), and musculoskeletal system (osteoarthritis, skeletal muscle atrophy, tendinopathies, spinal cord injury) [196,197]. Yet, such options are still in their infancy, facing a number of critical hurdles and challenges that need to be carefully addressed for the purpose of human disease control, including (1) ethical concerns, (2) the maintenance of mitochondrial integrity at isolation and upon storage prior to transplantation, (3) the choice of methodology for mitochondria delivery into the recipient as it may interfere with the efficiency of transplantation and lead to dissemination to non-target cells and tissues, (4) the viability and integrity of the transplanted mitochondria upon exposure to the extracellular environment (Ca^2+^ levels, temperature), (5) the compatibility of the transplanted mitochondria with the host nuclear DNA (and with the mtDNA) that may affect its functionality, expression, and retention, and (6) the cell source, dose, time point(s) and cycles of mitochondria administration to avoid a potential lack of sustained therapeutic effects [196,197].

In conclusion and as described in this review, despite the availability of advanced, promising strategies targeting the mitochondria to manage several prevalent, serious human disorders, a number of significant challenges remain to be carefully addressed for a future safe, effective, and practicable application of any of these approaches in clinical settings.

## Figures and Tables

**Table 1:** Factors involved in age-related human mitochondrial diseases.

Factors	Models	Mechanisms	Refs.
c-myc	aging cardiomyocytes	mitochondrial metabolism	[18]
PGC-1α	age-related pathologies (muscle, heart, liver, brain)	mitochondrial metabolism	[19,20]
Pim-1	myocardial infarction	calcium homeostasis, mitochondrial function	[18,21]
PUM2	aged nematodes, aged mouse muscle cells	mitochondrial dynamics, mitophagy	[22]
PFKFB3	cerebral ischemia-reperfusion injury	mitochondrial energy metabolism	[23]
ATF3	idiopathic pulmonary fibrosis	mitochondrial homeostasis	[24]
SIRT3	osteoporosis, cardiac hypertrophy	mitochondrial permeability, mitophagy	[25,26]
SIRT4	ionizing radiation aging	mitochondrial dynamics, mitophagy	[27]
NEAT1	chronic obstructive pulmonary disease	mitophagy	[28]
CoQ	heart and liver of aged mice	ETC	[29]
MitoQ	age-related endothelial dysfunction	oxidative damage to mitochondria	[30]
BGP-15	type 2 diabetes mellitus-associated cardiac dysfunction	ETC	[31]
GlcN	extends lifespan in nematodes and mice	mitochondrial metabolism	[32]
IP3R2	age-related liver fibrosis	MAMs	[33]
CISD2	premature aging	autophagy	[34]
isradipine	Parkinson’s disease	calcium uptake	[35]
verapamil	age-related hematopoietic dysfunction	age-related hematopoietic stem cell dysfunction	[36]
α-Klotho	regeneration of aging muscles	maintenance of mtDNA integrity, mitochondrial function	[37]
DRP1	extending the lifespan of Drosophila melanogaster	mitochondrial fission	[38]
FIS1	skeletal muscle aging	mitochondrial morphology	[39]
MIRO1	neuron disease	mitochondrial dynamics	[19]

Abbreviations: c-myc, cellular Myelocytomatosis; PGC-1α, peroxisome proliferator activated receptor gamma coactivator 1 alpha; Pim-1, proviral insertion in murine 1; PUM-2, pumilio2; PFKFB3, 6-phosphofructo-2-kinase/fructose-2,6-bisphosphatase 3; ATF3, activating transcription factor 3; SIRT3, sirtuin 3; SIRT4, sirtuin 4; NEAT1, nuclear enriched abundant transcript 1; CoQ, coenzyme Q; MitoQ, mitoquinone; BGP-15, O-(3-piperidino-2-hydroxy-1-propyl)nicotinic amidoxime; GlcN, D-glucosamine; IP3R2, inositol 1,4,5-triphosphate receptor 2; CISD2, CDGSH iron sulfur domain 2; DRP1, cytosolic dynamin-related protein 1; FIS1, fission protein-1; MIRO1, mitochondrial Rho 1; ETC, electron transport chain; MAMs, mitochondria-associated endoplasmic reticulum membranes.

## Data Availability

Not applicable.
